# Glutamate drives ‘local Ca^2+^ release’ in cardiac pacemaker cells

**DOI:** 10.1038/s41422-022-00693-z

**Published:** 2022-07-15

**Authors:** Duanyang Xie, Ke Xiong, Xuling Su, Guanghua Wang, Qicheng Zou, Luxin Wang, Caihong Zhang, Yuting Cao, Beihua Shao, Yixin Zhang, Peidong Zhang, Dandan Liang, Yi Liu, Yi-Han Chen

**Affiliations:** 1 Department of Cardiology, Shanghai East Hospital, Tongji University School of Medicine, Shanghai, China; 2 Key Laboratory of Arrhythmias of the Ministry of Education of China, Tongji University School of Medicine, Shanghai, China; 3 Institute of Medical Genetics, Tongji University, Shanghai, China; 4 Jinzhou Medical University, Jinzhou, Liaoning China; 5 Department of Pathology and Pathophysiology, Tongji University School of Medicine, Shanghai, China; 6 Research Units of Origin and Regulation of Heart Rhythm, Chinese Academy of Medical Sciences, Shanghai, China

**Keywords:** Cell biology, Molecular biology

## Abstract

The sinoatrial node (SAN) is the origin of the electrical signals for rhythmic heartbeats in mammals. The spontaneous firing of SAN pacemaker cells (SANPCs) triggers cardiac contraction. ‘Local Ca^2+^ release’ (LCR), a unique cellular activity, acts as the ‘engine’ of the spontaneous firing of SANPCs. However, the mechanism of LCR initiation remains unclear. Here, we report that endogenous glutamate drives LCRs in SANPCs. Using a glutamate sensor, we unraveled a tight correlation between glutamate accumulation and LCR occurrence, indicating a potential relationship between glutamate and LCRs. Intracellular application of glutamate significantly enhanced the LCRs in both intact and permeabilized SANPCs. Mechanistically, we revealed that mitochondrial excitatory amino acid transporter 1 (EAAT1)-dependent mitochondrial glutamate import promoted ROS generation, which in turn led to the oxidation of Ca^2+^-handling proteins, ultimately resulting in enhanced LCRs. Importantly, EAAT1 depletion reduced both the spontaneous firing rates of isolated SANPCs and the heart rate in vitro and in vivo, suggesting the central role of EAAT1 as a glutamate transporter in the regulation of cardiac autonomic rhythm. In conclusion, our results indicate that glutamate serves as an LCR igniter in SANPCs, adding a potentially important element to the coupled-clock theory that explains the origin of spontaneous firing. These findings shed new light on the future prevention and treatment of cardiac pacemaker cell-related arrhythmias.

## Introduction

The sinoatrial node (SAN) serves as the ‘headquarters’ of the heartbeat in mammals. Physiologically, SAN pacemaker cells (SANPCs) spontaneously and periodically generate electrical pulses to trigger heart contraction,^1–3^ and this process is termed spontaneous firing. ‘Local Ca^2+^ release’ (LCR) in SANPCs is generally accepted as the ‘engine’ of this spontaneous firing.^4–6^ LCRs generated by the sarcoplasmic reticulum (SR) are dominated by the Ca^2+^-handling proteins in SANPCs.^7,8^ Normally, among the Ca^2+^-handling proteins, ryanodine receptor 2 (RyR2) governed the SR Ca^2+^ release, SR Ca^2+^-ATPase 2a (SERCA2a) governed the SR Ca^2+^ uptake and together they shaped the LCRs.^9^ Phosphorylation and/or oxidation of these Ca^2+^-handling proteins can enhance LCRs in SANPCs.^10–14^ Glutamate, one of the most important excitatory transmitters in the nervous system, transmits information via the glutamate transmitter system (GluTS), including glutamate receptors, transporters and metabolic enzymes.^15,16^ It has been reported that GluTS mediates glutamate-induced currents and Ca^2+^ oscillations both in certain nerve and non-nerve cells.^17–19^ Recently, we identified endogenous GluTS in SANPCs and found that targeting the GluTS could effectively alter the frequencies of SANPC Ca^2+^ transients and SAN electrical pulses.^20^ These findings implied that glutamate plays a role in regulating Ca^2+^ dynamics in SANPCs.

The present study provides the first evidence that glutamate acts as an ‘igniter’ to ‘ignite’ LCR in SANPCs through a sequential biochemical process consisting of the mitochondrial excitatory amino acid transporter 1 (EAAT1)-dependent mitochondrial import of glutamate, increased reactive oxygen species (ROS) production, and oxidation of Ca^2+^-handling proteins. Our work adds a potentially important element to the coupled-clock theory, and the findings herein go beyond the conventional view that glutamate biologically functions as a neurotransmitter in the nervous system.

## Results

### Intracellular glutamate drives LCRs and spontaneous firing in rat SANPCs

**Fig. 1:**
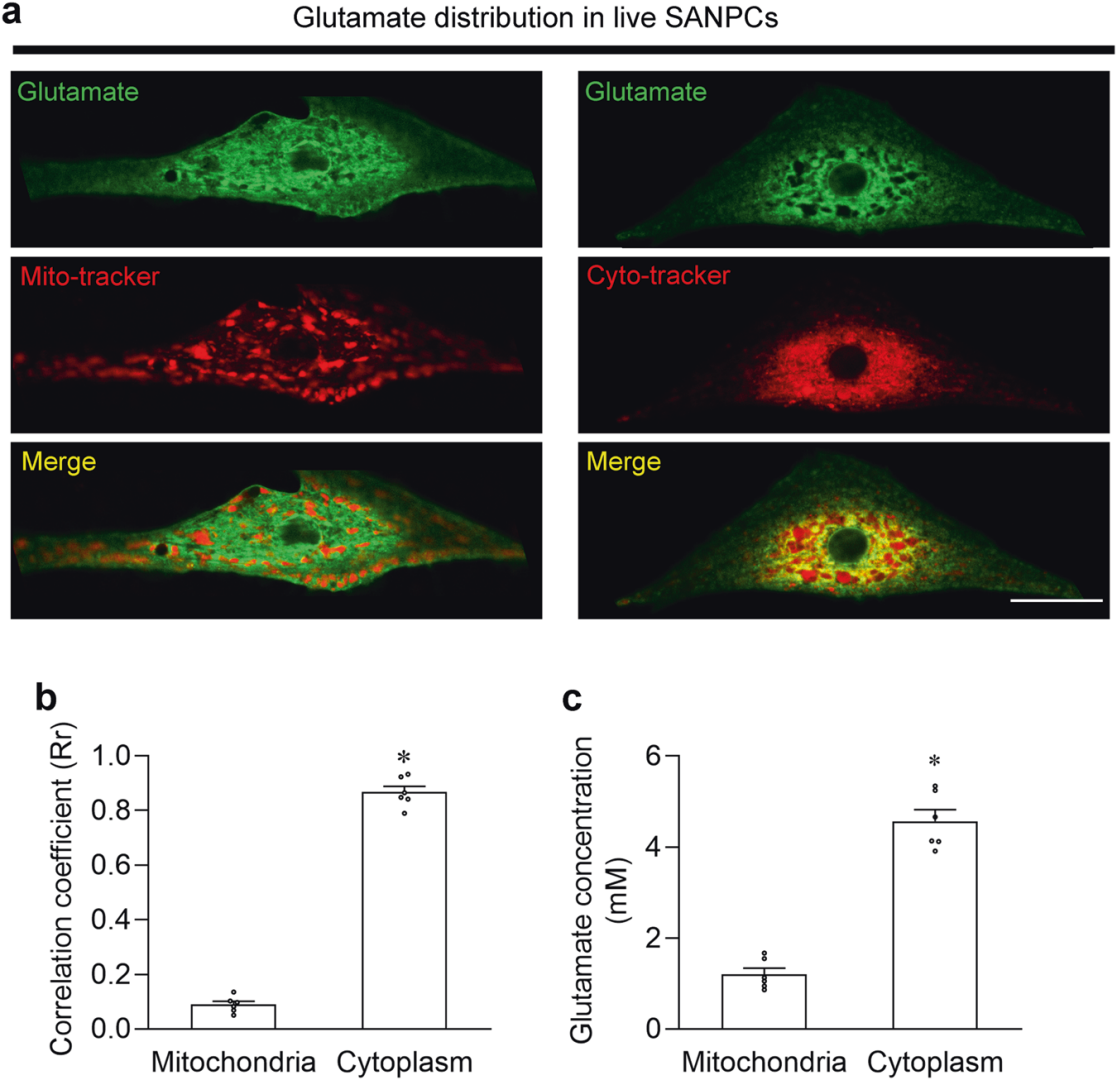
Glutamate was enriched in the cytoplasm but not in the mitochondria in rat SANPCs. **a** Representative confocal images of rat iGluSnFR-overexpressing SANPCs which were stained with Mito-tracker or Cyto-tracker. Scale bar = 20 µm. **b** Quantification of colocalization coefficients between glutamate and Mito-tracker/Cyto-tracker (*n* = 6). **c** Glutamate concentrations in mitochondrial and cytoplasmic subcellular components (*n* = 6). *P < 0.05, calculated by unpaired Student’s *t*-test.

First, we examined the subcellular distribution of glutamate in SANPCs. By utilizing a new type of intensity-based glutamate-sensing fluorescent reporter (iGluSnFR) and live-cell fluorescent probes (Mito-tracker probe for mitochondria and Cyto-tracker probe for the cytoplasm), we found that glutamate was more concentrated in the cytoplasm than in the mitochondria of SANPCs (Fig. 1a, b), which was further corroborated by immunofluorescence and biochemical analyses (Fig. 1c; Supplementary information, Fig. S1).

**Fig. 2:**
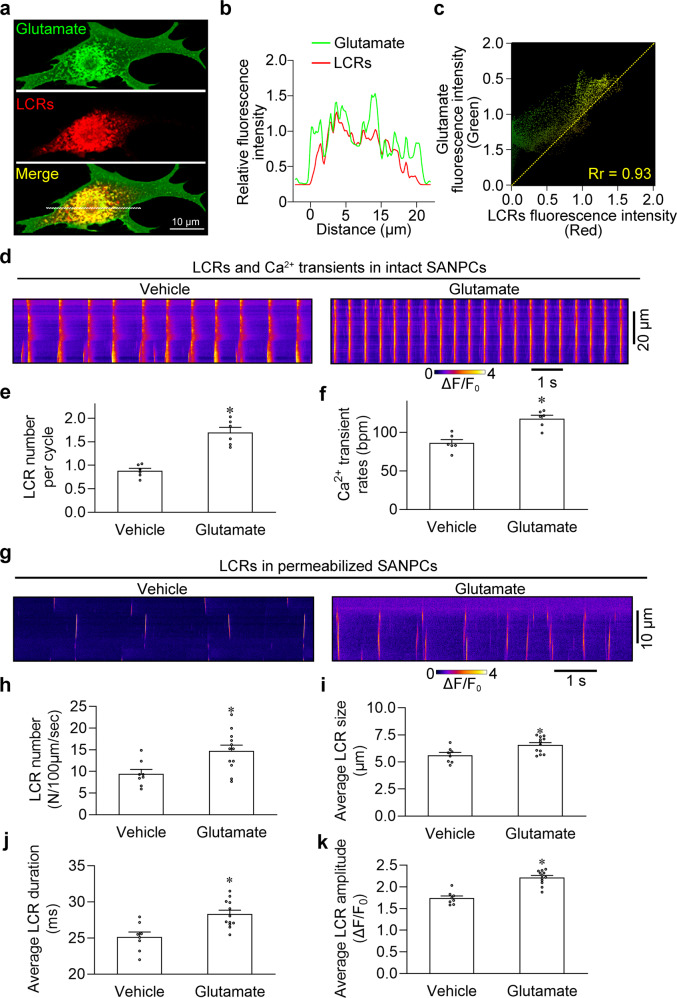
Colocalization of glutamate accumulation and LCR events in rat SANPCs. **a** Representative confocal images demonstrating the subcellular colocalization of glutamate accumulation and LCR events. **b** Line plot profiles of the fluorescence along the white dotted line in **a**. **c** Scatterplots showing the Pearson’s correlation coefficient (Rr) between the fluorescent signals of glutamate accumulation and LCR events. **d** Typical traces of spontaneous Ca^2+^ transients in SANPCs upon the intracellular injection of vehicle or 5 mM glutamate. **e**, **f** Pooled data from **d** demonstrated that glutamate application significantly increased the LCR number per cycle (**e**) and the Ca^2+^ transients rates (**f**) in SANPCs compared with the vehicle (*n* = 6 per group). **g** Representative confocal line-scan images of LCRs in permeabilized SANPCs. **h**–**k** The pooled data from **g** demonstrated that the application of 5 mM glutamate significantly increased LCR number (**h**), LCR size (**i**), LCR duration (**j**) and LCR amplitude (**k**) in permeabilized SANPCs compared to the vehicle group (*n* = 8–12 cells per group, cells were isolated from at least 5 rats). *P < 0.05, calculated by unpaired Student’s *t*-test.

Subsequently, we analyzed the association between glutamate and LCRs. Interestingly, synchronous dual imaging of glutamate and Ca^2+^ revealed that the distribution of glutamate was highly correlated with LCRs in SANPCs (Fig. 2a–c; Supplementary information, Video S1). In addition, we found that transient intracellular injection of glutamate increased both the number of LCRs and the spontaneous Ca^2+^ transient rate in SANPCs (Fig. 2d–f), indicating that glutamate regulates both the LCRs and the spontaneous firing in SANPCs.

We further investigated the effects of glutamate on LCRs in permeabilized SANPCs (Fig. 2g). The application of glutamate resulted in increases in the number, size, duration and amplitude of LCRs in permeabilized SANPCs (Fig. 2h–k), confirming that glutamate directly triggered LCRs independent of plasma membrane components of GluTS. Together, these results suggest that glutamate is mainly distributed in the SANPC cytoplasm rather than the mitochondria and that intracellular administration of glutamate enhances the LCR generation in SANPCs.

### Glutamate drives LCRs by governing the oxidation of Ca^2+^-handling proteins in SANPCs

**Fig. 3:**
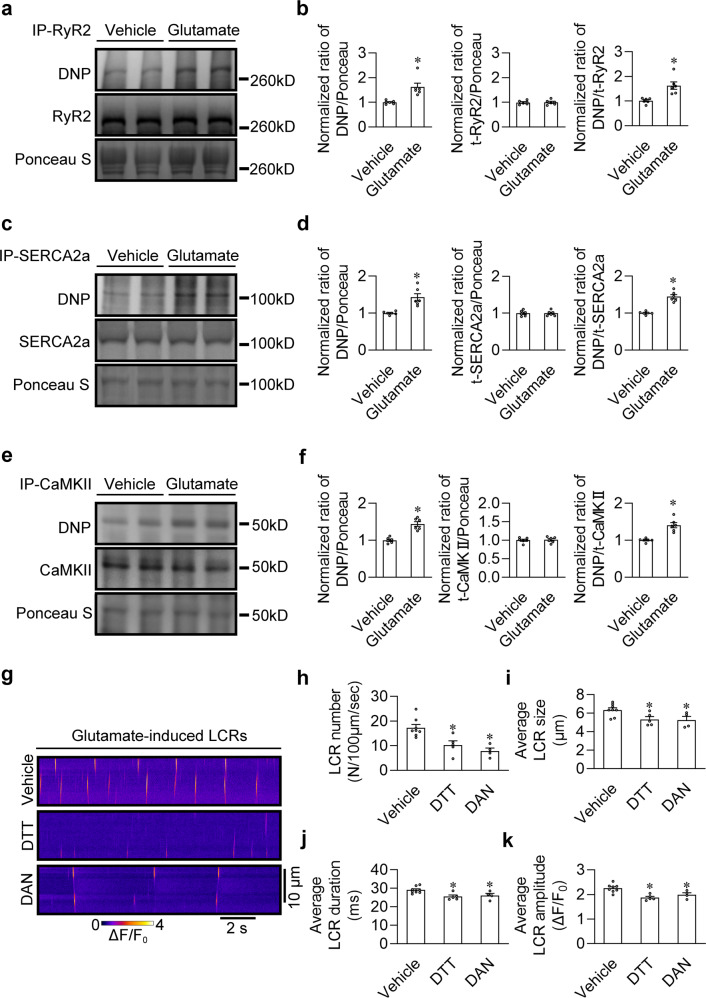
Glutamate triggered LCRs by increasing the oxidation of Ca^2+^-handling proteins. **a**, **b** To assess the oxidation status of RyR2, the free thiol content of immunoprecipitated RyR2 was measured using the anti-DNP antibody. Western blot showing increased oxidation of RyR2 under 5 mM glutamate treatment. **a** Representative western blot bands. **b** Pooled data from **a**. *n* = 6 per group. **c**, **d** To assess the oxidation status of SERCA2a, the free thiol content of immunoprecipitated SERCA2a was measured using the DNP antibody. Western blot showing increased oxidation of SERCA2a under glutamate treatment. **c** Representative western blot bands. **d** Pooled data from **c**. *n* = 6 per group. **e**, **f** To assess the oxidation status of CaMKII, the free thiol content of immunoprecipitated CaMKII was measured using the DNP antibody. Western blot showing increased oxidation of CaMKII under glutamate treatment. **e** Representative western blot bands. **f** Pooled data from **e**. *n* = 6 per group. **g** Representative confocal line-scan images of LCRs in permeabilized SANPCs. **h**–**k** Pooled data from **g** demonstrated that oxidation inhibitors (5 mM DTT or 10 μM dantrolene) reversed glutamate-induced increases in LCR number (**h**), LCR size (**i**), LCR duration (**j**) and LCR amplitude (**k**) in permeabilized SANPCs compared to the vehicle group (*n* = 4–8 per group, cells were isolated from at least 4 rats). DAN, dantrolene. *P < 0.05, calculated by unpaired Student’s * t*-test (**b**, **d**, **f**) or one-way ANOVA with Dunnett post-hoc test (**h**–**k**).

Glutamate is closely related to the intracellular oxidation state, and the oxidation of Ca^2+^-handling proteins significantly affects Ca^2+^ dynamics.^21,22^ Thus, we analyzed the effect of glutamate on the oxidation of Ca^2+^-handling proteins and the resulting LCRs. Since RyR2 and SERCA2a shape LCRs, while CaMKII regulates the function of RyR2 and SERCA2a,^4,5,10,23^ we analyzed the oxidation of these three Ca^2+^-handling proteins. As shown in Fig. 3a–f, administration of glutamate dramatically increased the oxidation of the three Ca^2+^-handling proteins without significantly changing their total protein level.

To confirm the role of Ca^2+^-handling protein oxidation in glutamate-induced LCR, we examined the effects of two antioxidants (DTT and dantrolene) on the glutamate-induced LCRs. We found that both antioxidants significantly decreased the number, size, duration and amplitude of LCRs (Fig. 3g–k). The above results indicate that the oxidation of Ca^2+^-handling proteins is crucial for the initiation of glutamate-induced LCRs in SANPCs.

### Glutamate-induced mitochondrial ROS increase mediates the oxidation of Ca^2+^-handling proteins in SANPCs

**Fig. 4:**
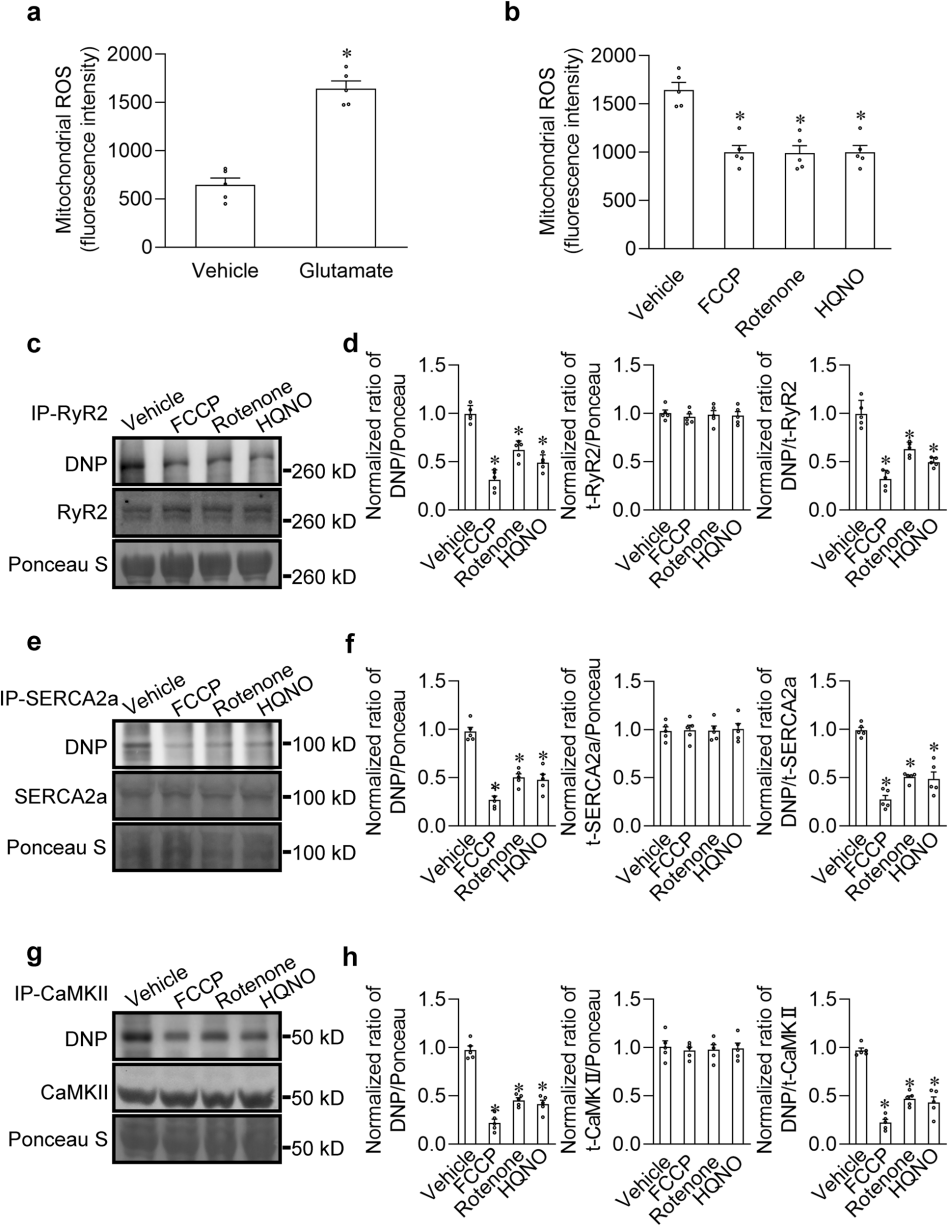
Glutamate-induced mitochondrial ROS generation promoted the oxidation of Ca^2+^-handling proteins in rat SANPCs. **a** Measurement of ROS levels in isolated SANPC mitochondria with or without 5 mM glutamate treatment (*n* = 5 per group). **b** Measurement of glutamate-induced ROS in SANPC mitochondria treated with vehicle or 10 µM FCCP, 10 µM rotenone or 10 µM HQNO (*n* = 5 per group). **c**–**h** To assess the oxidation status of RyR2, SERCA2a and CaMKII, the free thiol contents of these immunoprecipitated proteins were measured using the DNP antibody. Representative western blot images and semiquantitative analyses of glutamate-induced oxidation of RyR2 (**c**, **d**), SERCA2a (**e**, **f**) and CaMKII (**g**, **h**) treated with vehicle, 10 µM FCCP, 10 µM rotenone or 10 µM HQNO are shown. *P < 0.05, calculated by unpaired Student’s *t*-test (**a**) or one-way ANOVA with Dunnett post-hoc test (**b**, **d**, **f**, **h**).

It is well known that mitochondria are an important source of ROS in cells.^24^ Therefore, we hypothesized that the oxidation of Ca^2+^-handling proteins induced by intracellular application of glutamate may be primarily due to increased mitochondrial ROS production. As shown in Fig. 4a, glutamate treatment indeed increased the ROS production in the isolated mitochondria. Furthermore, the generation of ROS was effectively suppressed by the mitochondrial oxidative phosphorylation uncoupling agent (carbonyl cyanide 4-(trifluoromethoxy) phenylhydrazone, FCCP), mitochondrial complex I inhibitor (rotenone) and complex III inhibitor (2-heptyl-4-hydroxyquinoline-N-oxide (HQNO)) (Fig. 4b), indicating that the glutamate-induced ROS generation was mitochondria-dependent.

Next, we examined the effect of glutamate-induced mitochondrial ROS increase on the oxidation of Ca^2+^-handling proteins and LCRs, and found that FCCP, rotenone and HQNO all effectively downregulated the oxidation level of RyR2, SERCA2a and CaMKII (Fig. 4c–h) and reduced the number, size, duration and amplitude of LCRs (Supplementary information, Fig. S2). These results demonstrated that glutamate-induced mitochondrial ROS increase promotes the oxidation of Ca^2+^-handling proteins and LCRs in SANPCs.

### Mitochondrial EAAT1 mediates mitochondrial ROS increase in SANPCs

**Fig. 5:**
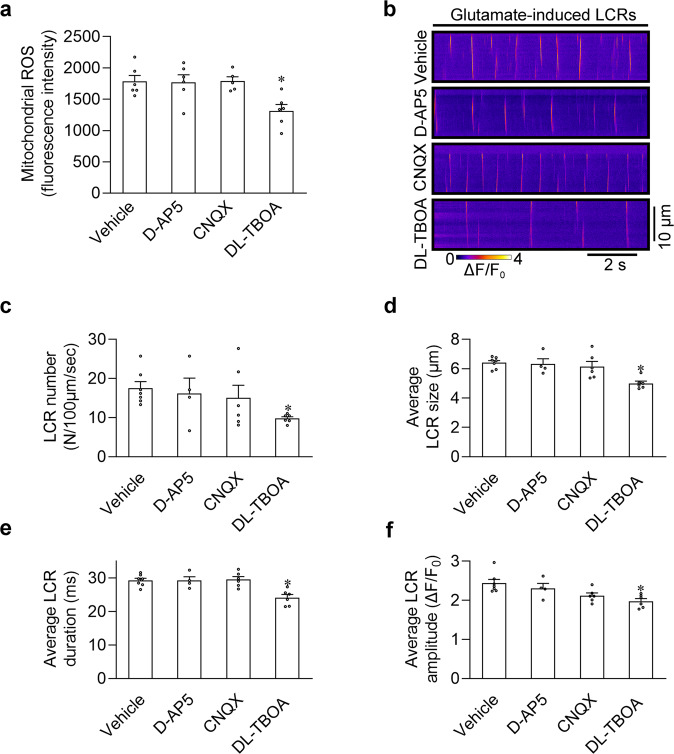
Glutamate transporters mediated glutamate-induced ROS production and LCR occurrence. **a** Measurement of 5 mM glutamate-induced ROS levels in rat SANPC mitochondria treated with vehicle or 100 μM inhibitors of glutamate receptors/transporters (*n* = 5–6 per group). **b** Representative confocal line-scan images of glutamate-induced LCRs in permeabilized SANPCs treated with vehicle or 100 μM D-AP5, CNQX and DL-TBOA. **c**–**f** Pooled data from **b** demonstrated that only DL-TBOA decreased the LCR number (**c**), LCR size (**d**), LCR duration (**e**) and LCR amplitude (**f**) compared with the vehicle (*n* = 4–7 per group, cells were isolated from at least 5 rats). *P < 0.05, calculated by one-way ANOVA with Dunnett post-hoc test.

**Fig. 6:**
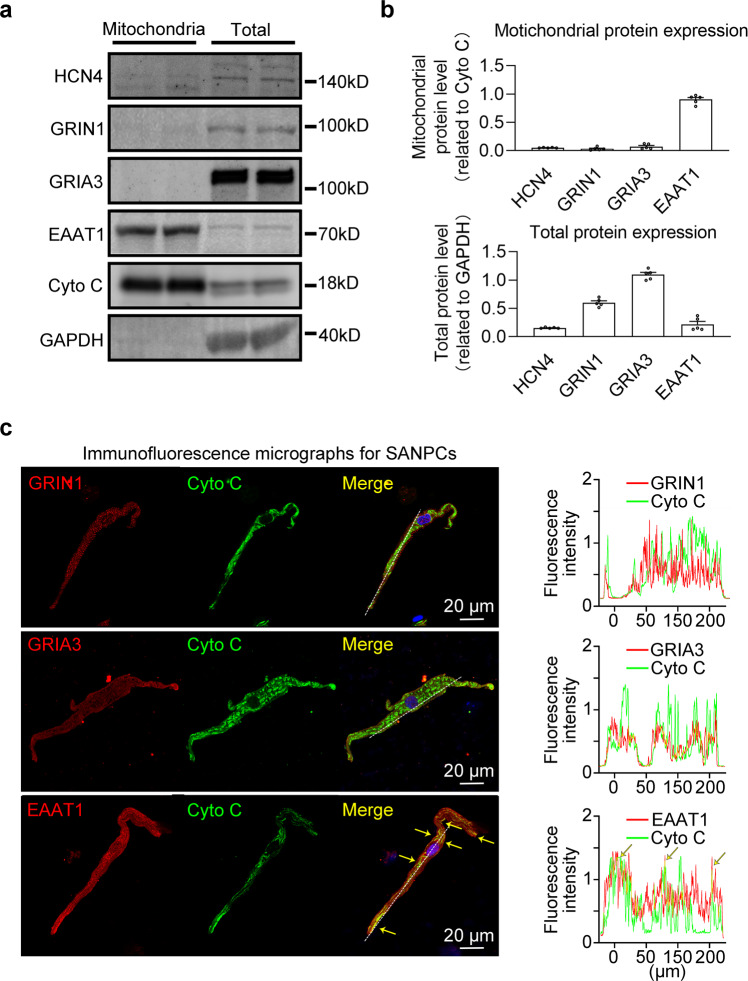
EAAT1 was abundantly expressed in rat SANPC mitochondria. **a** Western blot analysis confirmed that EAAT1 was abundantly expressed in SANPC mitochondria. **b** Pooled data from **a** (*n* = 5 per groups). **c** Immunofluorescence staining with anti-EAAT1 antibody, anti-GRIA3 antibody, anti-GRIN1 antibody and anti-Cytochrome C antibody (mitochondrial marker) in isolated SANPCs (left); line plot profiles of the fluorescence along the white dotted line on the left (right).

In general, glutamate functions through glutamate receptors and transporters; thus, we first assessed whether glutamate receptor inhibitors (CNQX for AMPA receptors and D-AP5 for NMDA receptors) and the glutamate transporter inhibitor (DL-TBOA for EAAT) can exert an effect on glutamate-induced mitochondrial ROS increase and LCRs in permeabilized SANPCs. After analyzing the effect of glutamate on LCRs, we isolated the mitochondria of SANPCs and measured the mitochondrial ROS content. The results showed that only the glutamate transporter inhibitor decreased glutamate-induced mitochondrial ROS accumulation and LCR enhancement of SANPCs (Fig. 5a–f), suggesting that these effects of glutamate in SANPCs may be mediated by glutamate transporters. Next, we performed proteomic analysis and identified that among glutamate transporters, only EAAT1 was present in SANPC mitochondria (Supplementary information, Fig. S3). Western blotting and immunofluorescence staining confirmed that EAAT1 was abundantly expressed in SANPC mitochondria (Fig. 6a–c).

### EAAT1 depletion attenuates the glutamate-induced mitochondrial ROS generation and automaticity of SANPCs

**Fig. 7:**
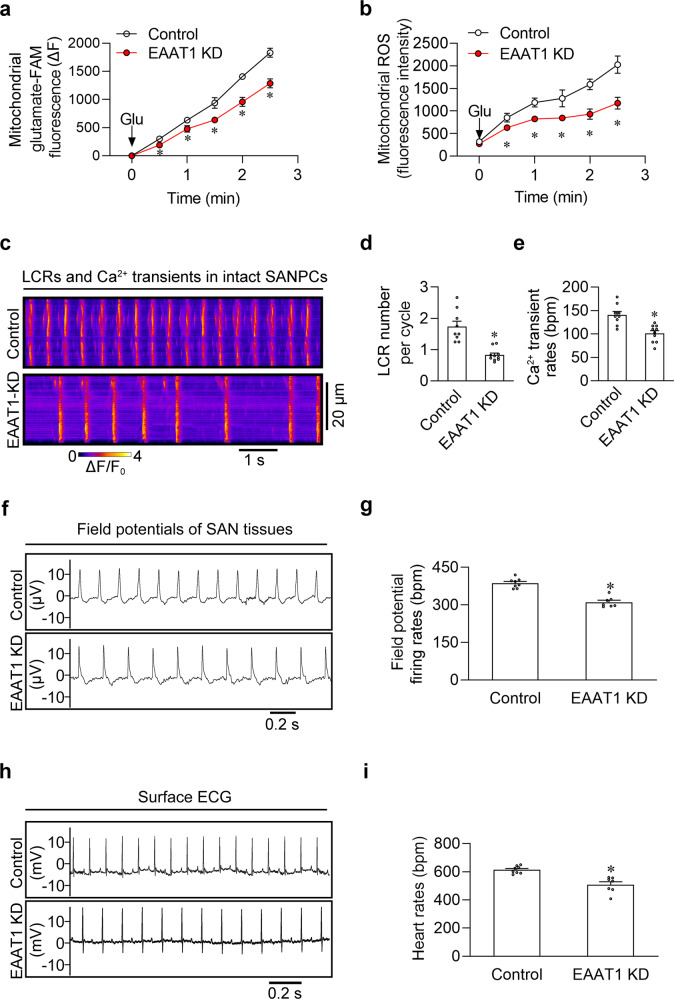
EAAT1 depletion attenuated mitochondrial glutamate import and automaticity in mouse SANPCs. **a** The mitochondrial FAM-glutamate import experiment showed that EAAT1 knockdown (EAAT1 KD) interrupted the time-dependent import of FAM-labeled glutamate into mitochondria (*n* = 4–6). **b** EAAT1 KD decreased glutamate-induced ROS production in SANPCs (*n* = 4–6). **c** Representative confocal line-scan images of spontaneous Ca^2+^ transients in SANPCs from the EAAT1 KD and control groups. **d**, **e** Pooled data from **c** (*n* = 9–10 per group). **f** Representative field potential recordings of SAN tissues from the EAAT1 KD and control groups. **g** Pooled data from **f** (*n* = 7–8 per group). **h** Representative surface ECG recordings of anesthetized mice from the EAAT1 KD and control groups. **i** Pooled data from **h** (*n* = 7–8 per group). *P < 0.05, calculated by unpaired Student’s *t*-test.

To evaluate the effects of EAAT1 depletion on mitochondrial ROS generation and automaticity of SANPCs, we knocked down EAAT1 in mouse SANPCs by using an adeno-associated virus serotype 9 (AAV9)-based strategy (Supplementary information, Fig. S4). As presented in Fig. 7a, b, EAAT1 depletion significantly restricted the mitochondrial import of glutamate and the mitochondrial ROS production in SANPCs. Additionally, confocal Ca^2+^ imaging detected the reduction in LCR frequency and spontaneous Ca^2+^ transient rates in EAAT1 knockdown SANPCs (Fig. 7c–e). Consistently, EAAT1 depletion also slowed down the spontaneous firing rates of SAN tissues and the heart rates of anesthetized mice (Fig. 7f–i). These results implicated that EAAT1 could be a potential target for the regulation of SAN spontaneous firing and the treatment of SANPC-related arrhythmia.

## Discussion

In the present study, we demonstrated that glutamate can trigger LCRs of SANPCs, which reveals that glutamate, a common neurotransmitter, plays a potentially important role in the initiation of spontaneous cardiac firing. We found that glutamate triggers LCRs via a mitochondrial EAAT1–mitochondrial glutamate import–ROS increase–oxidation of Ca^2+^-handling proteins axis, and the evidence is as follows. Firstly, we proved a tight correlation between glutamate accumulation and LCR occurrence. Secondly, intracellular application of glutamate resulted in LCR enhancement in both intact and permeabilized SANPCs. Thirdly, mitochondrial EAAT1-dependent mitochondrial glutamate import promoted ROS generation, resulting in oxidation of Ca^2+^-handling proteins and LCR enhancement. Finally, EAAT1 depletion slowed down spontaneous firing rates of SANPCs and heart rates.

Our findings showed that very low level of glutamate receptors was expressed in SANPC mitochondria and that interventions targeting glutamate receptors did not affect LCRs. The results implied that glutamate regulates the autonomic rhythm of SANPCs through a glutamate receptor-independent pathway. In contrast, EAAT1 was highly abundant in SANPC mitochondria, indicating that mitochondrial EAAT1 may mediate the biological efficacy of glutamate in SANPCs. Other studies also documented that EAAT1 can participate in mitochondrial glutamate transport in cardiomyocytes.^25^ Our data demonstrated that mitochondrial EAAT1 mediates mitochondrial import of glutamate and ROS production, triggering LCR and spontaneous Ca^2+^ transients, and ultimately altering pacing frequency of the SAN in vitro and in vivo. These results suggest that glutamate mainly affects the Ca^2+^ dynamics and pacemaker activity in SANPCs through mitochondrial EAAT1. Meanwhile, it is also possible that the EAAT1 serves as a potentially important target for the regulation of cardiac spontaneous firing and even for the intervention of cardiac pacemaker cell-associated arrhythmias.

Glutamate is an essential molecule for cell survival due to its critical role in cellular metabolism, we therefore cannot provide direct evidence for the proposition that without glutamate, the spontaneous firing of pacemaker cells would not occur. Nonetheless, we do provide positive data herein. On the one hand, glutamate application efficiently evoked large, fast and rhythmic LCRs, a precursor to the generation of spontaneous firing. On the other hand, the intracellular application of exogenous glutamate significantly increased the frequency of spontaneous firing in SANPCs.

Phosphorylation is another important modification for functional regulation of RyR2 and LCRs. Thus, we also examined the phosphorylation level of RyR2 upon glutamate stimulation in SANPCs (Supplementary information, Fig. S5). The result showed an enhanced phosphorylation in RyR2, suggesting that the enhanced phosphorylation may play a potential role in glutamate-induced LCR. However, whether the increased RyR2 phosphorylation is directly induced by glutamate or secondary to oxidative enhancement warrants further investigation. As our result showed, inhibition of Ca^2+^-handling protein oxidation can decrease the phosphorylation of RyR2, indicating that the phosphorylation of Ca^2+^-handling proteins might be an important intermediate link between the glutamate-induced ROS generation and LCRs in the SANPCs (Supplementary information, Fig. S6).

The spontaneous firing rate of the SAN mainly depends on two systems: the external autonomic nervous system and the inherent coupled-clock system.^1,5^ The external autonomic nervous system innervating the SAN consists of sympathetic and parasympathetic nerves; the former acts on adrenergic receptor on SANPCs via catecholamines, while the latter acts on cholinergic receptor on SANPCs via acetylcholine. The activation of both the adrenergic receptor and the cholinergic receptor ultimately regulates the autonomic rhythmic frequency of the SANPCs by affecting protein phosphorylation, thereby altering the functional activities of ion channels and the Ca^2+^ dynamics.^1,5^ However, importantly, the SAN triggers the heartbeat mainly through its own inherent autonomic rhythm, the origin of which is currently explained by the coupled-clock theory, and the external autonomic nervous system only serves as a regulatory factor. According to the coupled-clock theory, oscillation of the intracellular calcium concentration induced by LCR activates ion channels on the SANPC plasma membrane through the sodium–calcium exchange current, thus triggering periodic action potentials. The coupled-clock theory emphasizes the importance of the Ca^2+^-dependent activation of adenylate cyclase (AC). AC increases the cAMP concentration, thereby activating PKA, which together with Ca^2+^-activated CaMKII phosphorylates important components of the coupled-clock system, including the SR and plasma membrane proteins, and ultimately modifies cardiac spontaneous firing.^5^ In addition to the above two systems, other factors, such as neuropeptides, adenosine, hormones, mechanical load, atrial stretching, electrolytes and temperature, also participate in spontaneous cardiac firing modification.^1^ To the best of our knowledge, this study is the first to suggest that glutamate can drive LCRs through mitochondrial EAAT1 in SANPCs, complementing the coupled-clock theory from a small point.

In conclusion, our data confirm that intracellular endogenous glutamate can ignite LCRs in SANPCs. It should be emphasized that glutamate induces LCRs in SANPCs not through classical glutamate receptors but rather through mitochondrial glutamate transporter EAAT1. Mechanistically, endogenous glutamate triggers LCRs and the cardiac autonomic rhythm through mitochondrial EAAT1-dependent import of cytoplasmic glutamate into mitochondria, increasing ROS production, and oxidation of RyR2, SERCA2a and CaMKII. These findings may open new frontiers for studying the autonomic rhythm of cardiac pacemaker cells.

## Materials and methods

### Animals

This study was carried out in accordance with the Guide for the Care and Use of Laboratory Animals by the U.S. National Institutes of Health. All animal experiments were approved by the Animal Care and Use Committee of the Tongji University School of Medicine. All rats and mice were maintained under specific pathogen-free conditions. Male Sprague–Dawley rats and C57/BL6 mice were purchased from Shanghai Laboratory Animal Research Center.

### Isolation of SANPCs

SANPCs were isolated as previously described.^26,27^ Briefly, the rats or mice were anesthetized with intraperitoneal pentobarbital (25 mg/kg) or inhaled isoflurane (1.5% in 100% O_2_). The heart was quickly removed and placed in prewarmed Tyrode’s solution containing 140 mM NaCl, 5.4 mM KCl, 1.2 mM KH_2_PO_4_, 1.8 mM CaCl_2_, 1.0 mM MgCl_2_, 5.5 mM glucose and 5 mM HEPES (pH was adjusted to 7.4 with NaOH). The solution was continuously oxygenated with O_2_. The SAN was dissected from the region bordered by the crista terminalis and the superior and inferior vena cava. The SAN tissue was cut into small pieces and washed twice with low-Ca^2+^ solution containing 140 mM NaCl, 5.4 mM KCl, 1.2 mM KH_2_PO_4_, 0.2 mM CaCl_2_, 18.5 mM glucose, 50 mM taurine and 1.0% BSA (pH was adjusted to 6.9 with NaOH). The tissue was then digested at 36–37 °C for 20–25 min in a low-Ca^2+^ solution containing elastase (0.3 mg/mL; Worthington, NJ, USA), collagenase type IV (0.8 mg/mL; Sigma) and protease (0.13 mg/mL; Sigma, Chemical Co.) with gentle agitation. Digestion was terminated by transferring the pieces to modified Kraftbruhe (KB) solution containing 100 mM potassium glutamate, 10 mM potassium aspartate, 25 mM KCl, 10 mM KH_2_PO_4_, 2 mM MgSO_4_, 20 mM taurine, 5 mM creatine, 0.5 mM EGTA, 20 mM glucose, 5 mM HEPES and 1.0% BSA (pH was adjusted to 7.2 with KOH). For electrophysiological experiments, gradient recalcification of the acutely isolated cells was achieved via the application of Ca^2+^ solution at a final concentration of 1.8 mM at a 15-min interval.

### Primary culture of SANPCs and adenoviral infection

After gradient recalcification, the isolated SANPCs were centrifuged at 1000 × *g* for 3 min and resuspended in M199 medium supplemented with 10 mM 2,3-butanedione monoxime (Sigma, USA), 100× ITS-G (Gibco, USA), 0.1 mg/mL BSA (Sigma, USA), 0.1 μM isoprenaline and 1% Penicillin-Streptomycin (Gibco, USA). The cells were plated into Petri dishes or onto glass coverslips in the wells of cell culture plates. On the following day, the medium was replaced with fresh medium.

To express the glutamate sensor iGluSnFR in cultured SANPCs, adeno-CMV-iGluSnFR (packaged and supplied by Shanghai Taitool Bioscience Co. Ltd., China) was added to the medium (5 μL adenovirus/mL media, 1 × 10^10^ IFU/mL), and incubated with SANPCs for 8 h. Then, the medium was exchanged with fresh culture medium. Adenoviral infection was confirmed by visualizing iGluSnFR at 24 h post-infection. The iGluSnFR was excited with a 488-nm laser, and emission was detected at 500–540 nm.

### Immunofluorescence analysis

SANPCs were processed for immunofluorescence analysis with standard procedures. Briefly, the cells were fixed with fresh 4% paraformaldehyde for 15 min and washed twice with PBS for 5 min. The fixed cells were then permeabilized in PBS with 0.1% Triton X-100 for 10 min, blocked with 4% normal goat serum for 2 h at room temperature (RT) and incubated with primary antibodies against HCN4 (NeuroMab, USA), glutamate (Sigma, USA), EAAT1 (Abcam, USA), GAPDH (Proteintech, USA), Cytochrome C (Abcam, USA), GRIN1 (Abcam, USA) and GRIA3 (CST, USA) at 4 °C overnight. The cells were then washed with PBS with 0.1% Tween 20 (PBST) and incubated with secondary antibodies conjugated with Alexa Fluor (Abcam, USA) for 1 h at RT. After three washes with PBST, the nuclei were stained with DAPI.

### Isolation of mitochondria

Mitochondria were isolated from SANPCs with a Cell Mitochondria Isolation Kit (Beyotime, China) according to the manufacturer’s instructions. In brief, SANPCs were homogenized in a precooled mitochondrial separation reagent and centrifuged at 600 × *g* for 5 min at 4 °C. Subsequently, the supernatant was carefully collected and centrifuged at 11,000× *g* for 10 min at 4 °C, then pellet containing the isolated mitochondria was collected. Finally, the mitochondria were resuspended in a mitochondrial stock solution (Beyotime, China) for functional studies or mitochondrial lysate supplemented with protease inhibitor (cOmplete ULTRA Tablets, Roche, USA) for protein analysis.

### Glutamate concentration measurement

The glutamate concentrations in mitochondrial and cytoplasmic fractions were measured using a glutamate assay kit (Sigma, USA). The assay was based on enzymatic conversion, and the absorbance was read at 450 nm using a microplate reader (FlexStation3, Molecular Devices, USA). Then, glutamate production was measured according to the standard curve and the corresponding optical density.

### Ca^2+^ imaging

The Ca^2+^ in SANPCs was labeled with the Ca^2+^ indicator (AAT Bioquest, USA) Cal-590 (Ex/Em 555/580–625 nm) or Cal-520 (Ex/Em 488/500–540 nm), and 2D images of Ca^2+^ dynamics were acquired using the STELLARIS ultrafast confocal system (Leica, USA) with a 63× oil objective (NA 1.4). A line scan of Ca^2+^ dynamics was obtained with an Sp8 confocal microscope (Leica, USA) and a 63× oil objective (NA 1.4) at 35 ± 0.5 °C. To determine the effects of transient intracytoplasmic injection on SANPCs, glutamate or vehicle was injected via a glass micropipette fitted to a PL1–100 picoinjector (Harvard Apparatus, USA).

LCR events in permeabilized SANPCs were measured as described previously.^4^ Isolated SANPCs were perfused with an internal solution (50 nM free [Ca^2+^]_i_) containing 0.5 mM EGTA, 20 mM HEPES, 100 mM potassium aspartate, 25 mM KCl, 10 mM NaCl, 0.81 mM MgCl_2_, 3 mM ATP-Mg, and 10 mM phosphocreatine with 5 U/mL creatine phosphokinase and 0.01% saponin for 45 s for membrane permeabilization and then perfused with an internal solution containing 6 µM Cal-520, potassium salt. The LCR number was determined from the line scans, and the LCR amplitude was defined as the peak fluorescence over the background fluorescence (ΔF/F_0_). The LCR size (in micrometers) was indexed as the full width at half maximum amplitude (range from 3 to 20 μm), and the LCR duration (in milliseconds) was characterized as the full duration at half maximum amplitude (range from 15 to 60 ms).

### Western blotting and Immunoprecipitation

Proteins were purified from SANPCs as described in previous studies.^28^ Briefly, equal amounts of protein were separated by 10% SDS–PAGE (Thermo Fisher Scientific, USA) and then transferred onto PVDF membranes (Millipore, USA). Next, the membranes were blocked with 5% nonfat milk in 0.1% Tween wash buffer for 1 h at RT and then incubated with a primary antibody overnight at 4 °C. The antibodies used in this study were as follows: anti-Dinitrophenyl (DNP), anti-RyR2 (Abcam, USA), anti-SERCA2 ATPase (Abcam, USA), anti-CaMKII (Abcam, USA) anti-EAAT1 (Abcam, USA), anti-Cytochrome C (Abcam, USA), anti-GAPDH (Proteintech, USA), anti-GRIN1 (Abcam, USA), anti-HCN4 (NeuroMab, USA) and anti-GRIA3 (CST, USA). The next day, the membranes were incubated with HRP-conjugated secondary antibody for 1 h at RT, washed and detected with enhanced chemiluminescence reagent. On the bands were visualized using a ChemiDoc Touch Gel imaging system (Bio–Rad, USA).

Immunoprecipitation (IP) was performed by incubating protein lysate with anti-RyR2 (Abcam, USA), anti-SERCA2a (Abcam, USA), anti-CaMKII (Abcam, USA) antibodies in 1 mL IP buffer, respectively, on a microtube rotator at 4 °C overnight. Protein G-Sepharose beads (Thermo, USA) were added to each sample, and incubated for 3 h. After centrifugation for 1 min at 1000× *g*, the pellets were washed with IP buffer four times, and then supplemented with 5× sample buffer. Samples were incubated at 99 °C for 5 min and Western blot assay was carried out. The oxidation of RyR2, SERCA2a or CaMKII was evaluated by detecting the free thiol contents using the DNP antibody in the IP samples as previously described.^29^ All the images of the western blots were analyzed using the ImageJ (NIH) analysis software.

### Measurement of ROS

ROS production in mitochondria isolated from SANPCs was measured with the DCF ROS/RNS Assay Kit (Abcam, USA) according to the manufacturer’s protocol. In brief, the isolated mitochondria were homogenized in cold PBS and centrifuged at 10,000× *g* for 5 min. The supernatant was collected for the assay, and fluorescence (Ex/Em 480/530 nm) was measured using a microplate reader (FlexStation3, Molecular Devices, USA).

### Identification of mitochondrial proteins by SDS–PAGE and mass spectrometry

Mitochondrial lysates were separated by 10% SDS–PAGE, and the bands were excised from the gel for mass spectrometry (MS) sequence analysis. LC-MS/MS was performed by Shanghai Applied Protein Technology Co., Ltd. (Shanghai, China) with a Q Exactive mass spectrometer coupled to Easy-nLC (Thermo Fisher Scientific, USA) using a routine method. Spectral data were searched against a UniProt rat database (www.uniprot.org), and a protein–protein interaction (PPI) network was constructed using the STRING database (https://www.string-db.org/).

### In vitro mitochondrial import assay

An in vitro mitochondrial import assay was performed using freshly isolated mitochondria and 5-FAM-labeled glutamate (SciLight Peptide, China). The fluorescence of 5-FAM was used to measure glutamate uptake in mitochondria. Briefly, 5-FAM-labeled glutamate was incubated with mitochondria at RT for various time points in a mitochondrial stock solution (Beyotime, China). Then, the mitochondria were thoroughly washed to remove all exogenous fluorescent glutamate, and the fluorescence intensity of 5-FAM (Ex/Em 492/518 nm) was measured by a microplate reader (FlexStation3, Molecular Devices, USA).

### AAV generation and AAV9 virus injection

The *Slc1a3*-targeting miRs were designed using Invitrogen’s online software (Invitrogen’s RNAi Designer: http://rnaidesigner.thermofisher.com/rnaiexpress/design.do; Target Design Options: miR RNAi). According to the software, miRNA sequences targeting the different positions of the *Slc1a3* gene (*Slc1a3*-miRNA) and negative control sequence (Control-miRNA) were designed, synthesized, and cloned into the pAAV9-mCherry-miRNA plasmid vectors with the *Hcn4* promoter. The pAAV9-mCherry-miRNA (*Slc1a3*) and pAAV9-mCherry (Control) plasmids were constructed using BLOCK-iT Pol II miR RNAi Expression Vector Kits (Invitrogen, USA). The validated miRNA sequences (target sequence for *Slc1a3*-miRNA: CCTCACCAAGGAAGATGTTAA; target sequence for control-miRNA: GTCTCCACGCGCAGTACATTT) were packaged into viral vectors. The virus was then injected into adult (6–8 weeks old) male mice through the tail vein (2 × 10^11^ viral particles/mouse). AAV9 infection was confirmed by visualizing the expression of mCherry under fluorescence microscopy two weeks after infection, and the effectiveness was further confirmed in mouse SAN tissues by western blotting.

### Field potential recordings of SAN tissues

SAN tissues from the mice were dissected and placed in an oxygenated Tyrode’s solution at 37 °C. Field potential recordings were acquired as previously described.^20^ Two shielded Ag/AgCl electrodes were positioned at the proximal regions of the superior and inferior vena cava to record the field potential. The tissues were equilibrated in the tissue bath until electrically stable. The electrical signals were amplified, digitized, and visualized during the experiment using LabChart7 (ADInstruments, USA).

### Surface electrocardiogram (ECG)

Surface ECG recordings were obtained in anesthetized mice using the Powerlab system and BioAmp (ADInstruments, USA). Three-needle ECG electrodes were positioned subcutaneously, and the ECG was recorded from lead II. Recordings were observed, analyzed, and quantified using Labchart software (ADInstruments, USA).

### Statistical analyses

Each animal sample and every chemical used in the electrophysiological experiments was assigned a code, and the data were masked until completion of the study. Experiments were performed at least for three times, with independent samples. Data normality was assessed using the Kolmogorov–Smirnov test. For comparisons between two groups, two-tailed Student’s *t*-test was used. One-way ANOVA was used for multiple-group comparisons. All the data are presented as the means ± SEM. All statistical analyses were performed with GraphPad Prism 8.

## Supplementary information

Supplementary information, Figure S1

Supplementary information, Figure S2

Supplementary information, Figure S3

Supplementary information, Figure S4

Supplementary information, Figure S5

Supplementary information, Figure S6

Supplementary information, Video S1

Supplementary information, Video Legend
