# Supplementary material for: Glutamate drives ‘local Ca2+ release’ in cardiac pacemaker cells
Source: Cell Res. 2022 Jul 15;32(9):843–54. doi: 10.1038/s41422-022-00693-z (PMC9437105; doi:10.1038/s41422-022-00693-z)
Supplement: Supplementary file 1 — Supplementary information, Figure S1 [file 41422_2022_693_MOESM1_ESM.pdf]

Supplementary Figures

Fig. S1

a

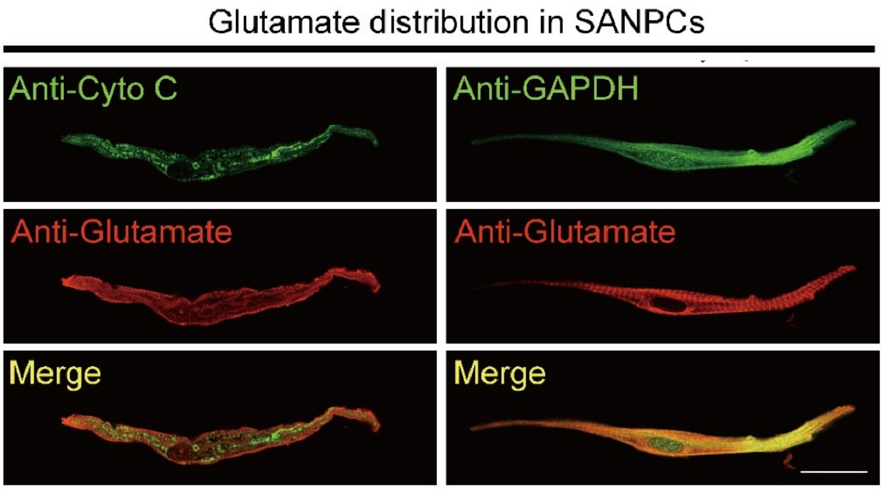

b

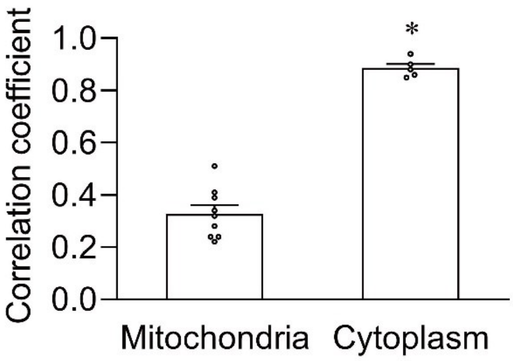

Fig. S1. Glutamate was more present in the cytoplasm than in the mitochondria in rat SANPCs.

a Immunofluorescence staining with anti-glutamate, anti-Cytochrome C (mitochondrial marker) and anti-GAPDH (cytoplasmic marker) antibodies in isolated SANPCs. Scale bar = 20 μm. **b** Quantification of the colocalization of glutamate with Cytochrome C or GAPDH (n = 5-9 per group; * $p < 0.05$, p values were calculated by unpaired Student's t-test). Cyto C, cytochrome C.
