# Supplementary material for: Glutamate drives ‘local Ca2+ release’ in cardiac pacemaker cells
Source: Cell Res. 2022 Jul 15;32(9):843–54. doi: 10.1038/s41422-022-00693-z (PMC9437105; doi:10.1038/s41422-022-00693-z)
Supplement: Supplementary file 2 — Supplementary information, Figure S2 [file 41422_2022_693_MOESM2_ESM.pdf]

Fig. S2

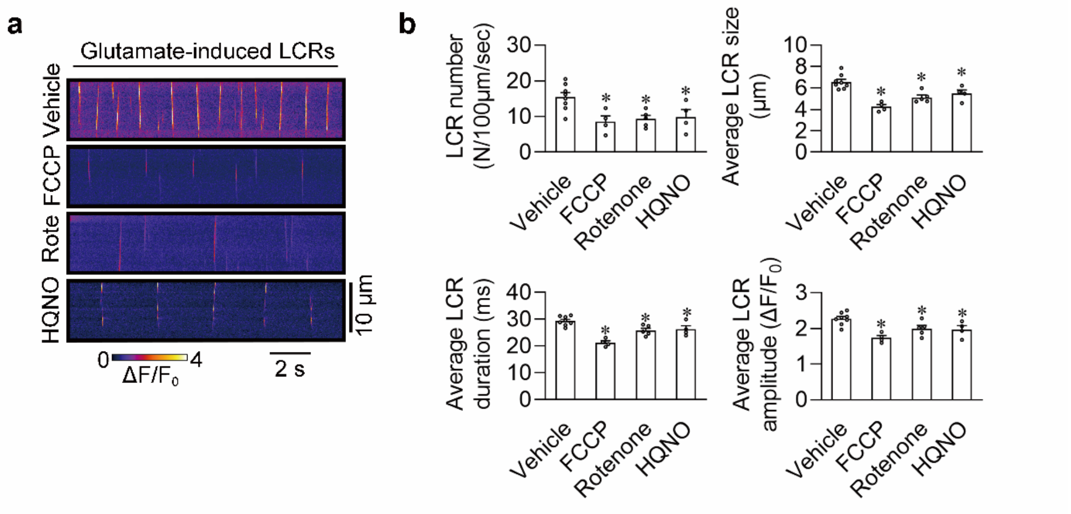

Fig. S2. Glutamate-induced mitochondrial ROS increase mediated the LCR enhancement in rat SANPCs.

a Representative confocal line-scan images of LCR events in 5 mM glutamate-treated, permeabilized SANPCs treated with vehicle or 10 μM FCCP, 10 μM Rotenone or 10 μM HQNO. **b** Pooled data from **a** (n = 4-8 cells per group, cells were isolated from at least 4 rats; * $p < 0.05$, p values were calculated by one-way ANOVA with Dunnett post-hoc test). Rote, Rotenone.
