# Supplementary material for: Glutamate drives ‘local Ca2+ release’ in cardiac pacemaker cells
Source: Cell Res. 2022 Jul 15;32(9):843–54. doi: 10.1038/s41422-022-00693-z (PMC9437105; doi:10.1038/s41422-022-00693-z)
Supplement: Supplementary file 3 — Supplementary information, Figure S3 [file 41422_2022_693_MOESM3_ESM.pdf]

Fig. S3

a

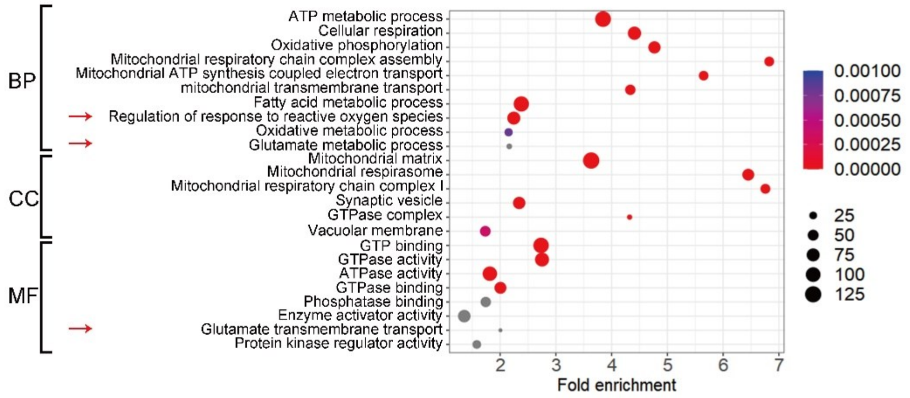

b

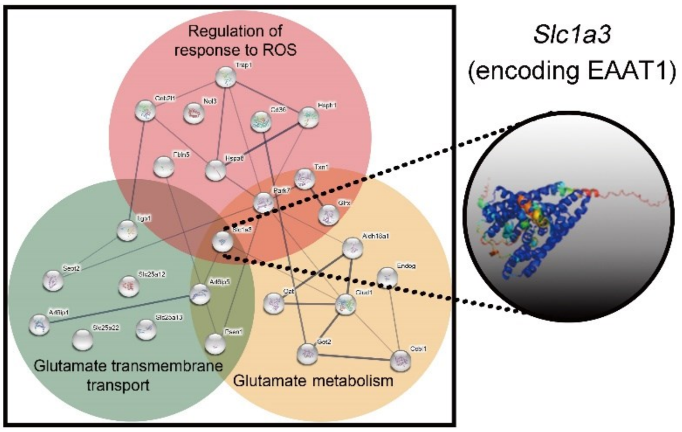

Fig. S3. Identification of the glutamate transporter EAAT1 in rat SANPC mitochondria.

a Gene ontology analysis of SANPC mitochondria. The significantly enriched pathways involving the glutamate metabolism and reactive oxygen species were indicated (red arrows). **b** Protein-protein interaction (PPI) network of SANPC mitochondrial proteins built using STRING. The analysis highlighted the expression of EAAT1 in SANPC mitochondria. BP, Biological Process; CC, Cellular Component; MF, Molecular Function.
