# Supplementary material for: Glutamate drives ‘local Ca2+ release’ in cardiac pacemaker cells
Source: Cell Res. 2022 Jul 15;32(9):843–54. doi: 10.1038/s41422-022-00693-z (PMC9437105; doi:10.1038/s41422-022-00693-z)
Supplement: Supplementary file 4 — Supplementary information, Figure S4 [file 41422_2022_693_MOESM4_ESM.pdf]

Fig. S4

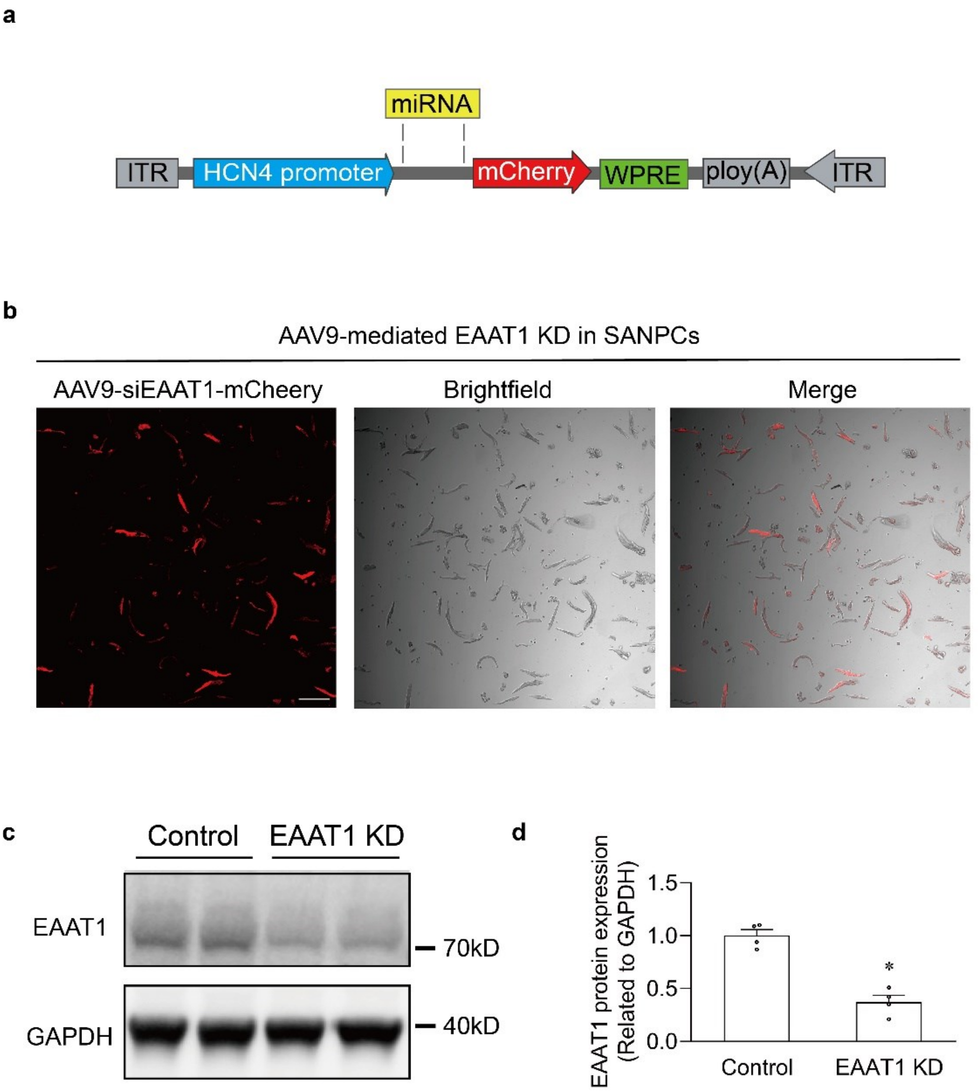

Fig. S4. The efficiency of EAAT1 knockdown by adeno-associated virus-9 (AAV9)-based strategy.

a Schematic diagram showed the construction of AAV9 virus under the control of HCN4 promoter. **b** Fluorescence imaging of SANPCs isolated from mice 2 weeks after AAV9 virus injection. Scale bar = 100 μm. **c** Representative western blot bands showed the efficiency of EAAT1 knockdown (EAAT1 KD) by AAV9-siEAAT1 in mice. **d** Quantitative analysis for **c** (n = 4; * $p < 0.05$, p values were calculated by unpaired Student's t-test).
