# Supplementary material for: Glutamate drives ‘local Ca2+ release’ in cardiac pacemaker cells
Source: Cell Res. 2022 Jul 15;32(9):843–54. doi: 10.1038/s41422-022-00693-z (PMC9437105; doi:10.1038/s41422-022-00693-z)
Supplement: Supplementary file 5 — Supplementary information, Figure S5 [file 41422_2022_693_MOESM5_ESM.pdf]

Fig. S5

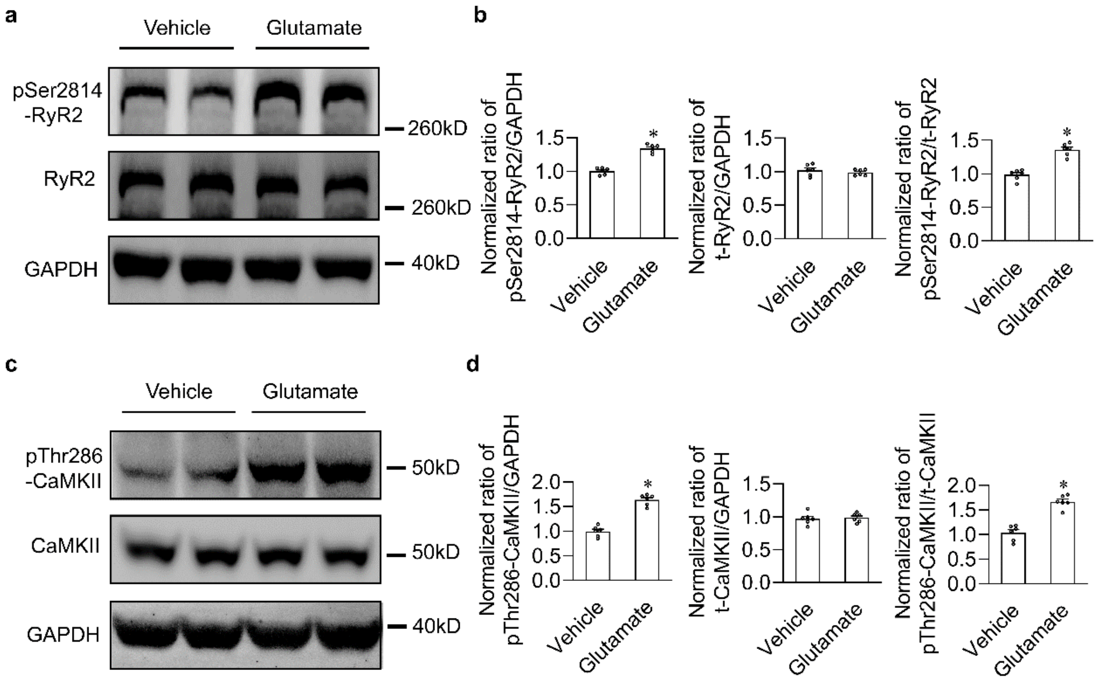

Fig. S5. Glutamate increased the phosphorylation of Ca^{2+}-handling proteins in rat SANPCs.

a-b Western blot showing that increased phosphorylation of RyR2 (Ser-2814) in SANPCs with 5 mM glutamate treatment. **a** Representative western blot bands. **b** Pooled data from **a**. n = 6 per group. **c-d** Western blot showing that increased phosphorylation of CaMKⅡ (Thr-286) in SANPCs with glutamate treatment. **c** Representative western blot bands. **d** Pooled data from **c**. n = 6 per group. * $p < 0.05$, *p* values were calculated by unpaired Student's *t*-test.
