# Supplementary material for: Glutamate drives ‘local Ca2+ release’ in cardiac pacemaker cells
Source: Cell Res. 2022 Jul 15;32(9):843–54. doi: 10.1038/s41422-022-00693-z (PMC9437105; doi:10.1038/s41422-022-00693-z)
Supplement: Supplementary file 6 — Supplementary information, Figure S6 [file 41422_2022_693_MOESM6_ESM.pdf]

Fig. S6

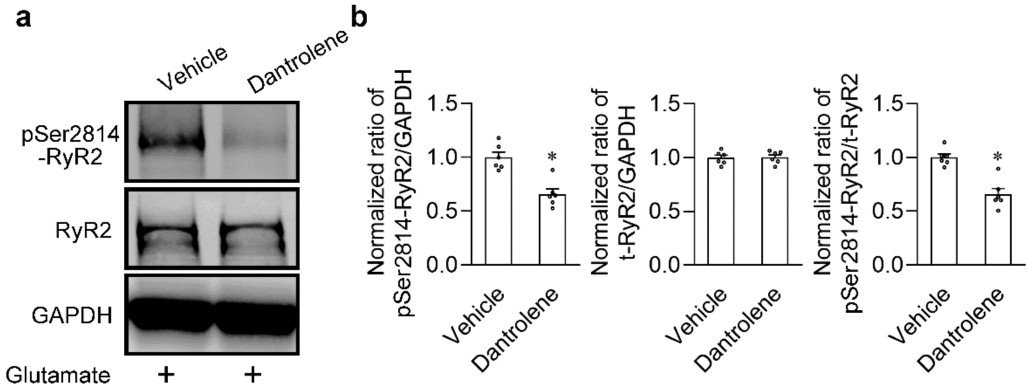

Fig. S6. Dantrolene decreased the glutamate-induced RyR2 phosphorylation in rat SANPCs.

Western blot showing that glutamate-induced RyR2 phosphorylation (Ser-2814) was decreased by 10 μM Dantrolene in SANPCs. **a** Representative western blot bands. **b** Pooled data from **a**. n = 6 per group. * $p < 0.05$, calculated by unpaired Student's *t*-test. t-RyR2, total RyR2.
