# Supplementary material for: Glutamate drives ‘local Ca2+ release’ in cardiac pacemaker cells
Source: Cell Res. 2022 Jul 15;32(9):843–54. doi: 10.1038/s41422-022-00693-z (PMC9437105; doi:10.1038/s41422-022-00693-z)
Supplement: Supplementary file 8 — Supplementary information, Video Legend [file 41422_2022_693_MOESM8_ESM.pdf]

Supplementary Video Legend

Video S1. Video of the synchronously recording for glutamate accumulation (green) and LCR events (red) in single SANPC.
